# Comparison of different NAT assays for the detection of microorganisms belonging to the class *Mollicutes*

**DOI:** 10.1186/s12917-017-1116-2

**Published:** 2017-06-24

**Authors:** O. Vega-Orellana, J. B. Poveda, R. S. Rosales, J. M. Bradbury, C. G. Poveda, L. E. Mederos-Iriarte, M. M. Tavío, A. S. Ramírez

**Affiliations:** 1 Unidad de Epidemiología y Medicina Preventiva, Facultad de Veterinaria, Universidad de Las Palmas de Gran Canaria, C/Trasmontaña s/n, 35413 Arucas, Gran Canaria Spain; 2 University of Liverpool, School of Veterinary Science, Leahurst Campus, Neston, CH64 7TE UK; 3 Microbiología, Departamento de Ciencias Clínicas, Facultad de Ciencias de la Salud, Universidad de Las Palmas de Gran Canaria, Las Palmas de Gran Canaria, Spain

**Keywords:** Mollicutes, Mycoplasma, Diagnostic, Nat, PCR, Real-time PCR

## Abstract

**Background:**

Mollicutes detection can be cumbersome due to their slow growth in vitro. For this reason, the use of DNA based on generic molecular tests represents an alternative for rapid, sensitive and specific detection of these microorganism. For this reason, six previously described nucleic acid testing assays were compared to evaluate their ability to detect microorganisms belonging to the class *Mollicutes*.

**Methods:**

A panel of 61 mollicutes, including representatives from the *Mycoplasma*, *Acholeplasma*, *Mesoplasma*, *Spiroplasma* and *Ureaplasma* genus, were selected to evaluate the sensitivity and specificity of these assays. A total of 21 non-mollicutes, including closely related non-mollicutes species, were used to evaluate specificity. Limits of detection were calculated to determine the analytical sensitivity of the assays. The two best performing assays were subsequently adapted into real-time PCR format, followed by melting curve analysis.

**Results:**

Both assays performed satisfactorily, with a 100% specificity described for both assays. The detection limits were found to be between 10^−4^ and 10^−5^ dilutions, equivalent to 15 to 150 genome copies approximately. Based on our work, both van Kuppeveld and Botes real-time PCR assays were found to be the best performing tests in terms of sensitivity and specificity. Furthermore, Botes real-time PCR assay could detect phytoplasmas as well.

**Conclusions:**

These assays can be very useful for the rapid, specific and sensitive screening cell line contaminants, clinical samples as well as detecting non-culturable, unknown species of mollicutes or mollicutes whose growth is slow or difficult.

## Background

Mollicutes are the smallest self-replicating free-living microorganisms. This class contains nine genera: *Mycoplasma, Ureaplasma, Entomoplasma, Mesoplasma, Spiroplasma, Acholeplasma*, “Candidatus *Phytoplasma*”, *Anaeroplasma* and *Asteroleplasma*. Genus *Mycoplasma* includes organisms transferred from genus *Haemobartonella* and genus *Eperythrozoon*, which are haemotropic mycoplasmas [1].

Mollicutes are considered commensal or parasite bacterial species. Many members of this bacterial class are significant pathogens of human, animals, insects and plants [1], including the aetiological agents of various World Organisation for Animal Health (OIE) listed diseases, such as contagious bovine pleuropneumonia, contagious agalactia, contagious caprine pleuropneumonia and avian mycoplasmosis. In addition, mycoplasma contamination of cell lines is by far the most frequently occurring problem in cell culture and biologic manufacturing processes [2, 3].

Culture isolation is essential for a definitive identification of the organism. However, mollicutes can be fastidious to isolate in vitro and difficult to identify phenotypically [1]. Another conventional method used for the diagnosis of infections caused by mollicutes are serological tests, however they can be time-consuming and can lack of sensitivity and specificity [4], as they rely on specific seroconversion. Methods based on the use of nucleic acid amplification technology (NAT) for detecting mollicutes have been used since the 1980s and have been successfully applied in the fields of animal health, human health and the control of contaminants in cell cultures [5–7]. Besides, recommendations for evaluation of NAT assays for mollicutes testing were included in European Pharmacopoeia monograph 2.6.7 [7, 8] as well as in other pharmacopoeias.

NAT techniques such as polymerase chain reaction (PCR) have attracted much attention due to its extreme sensitivity and specificity. These techniques have been widely applied for mollicutes determination [5, 9]. They also improve the efficiency of mollicutes detection [7] because of their simplicity, reduced testing time in comparison to traditional detection systems applied to biological products and diagnostic samples. However, some PCR methods are not sufficiently sensitive to detect the many mollicutes necessary for replacing the general culture-based tests [3, 10]. Recently, real-time PCR protocols are starting to be adapted and developed for the detection of some mollicutes. This type of PCR has a higher sensitivity in comparison to conventional PCR. It also produces quicker results and can quantify DNA in tested samples [11, 12].

PCR methods targeting different genetic markers of mollicutes have been previously developed and applied for species-specific of these microorganisms. However, when the aim is to detect several mollicutes species simultaneously, the PCR should be based on highly conserved regions, such as the 16S ribosomal RNA (rRNA) gene [6, 13, 14], the adjacent regions of the 16S–23S rDNA intergenic space region (ISR) [15, 16] or the 23S rDNA [17]. A single generic test that enables simultaneous mycoplasma identification to the species level, based on a PCR-DGGE, has also been described [18]. More recently, DNA microarray assays for mycoplasma species identification have been published [19–21], however their availability worldwide is still limited.

The aim of this study was to compare the sensitivity and specificity of six published PCR assays for the detection of mollicutes, to determine the most suitable assay for mollicutes detection. Additionally, a SYBR Green-based real-time PCR assay was optimised, after evaluating the two best performing conventional PCR assays, for a faster detection of mollicutes.

## Materials and methods

### Organisms and culture conditions

Sixty reference strains and field samples of the class *Mollicutes* and DNA from a *Mycoplasma (M.) wenyonii* positive sample were used in this study (Table 1). Mollicutes were growth in SP4-II broth [22] from 2 to 5 days in normal atmospheric conditions at 37 °C, while *Mesoplasma* (*Me*.) *florum*, *Acholeplasma* and *Spiroplasma* spp. were growth at 25 °C. All samples were filter-cloned [23] to ensure purity.

**Table 1:** Results of six different PCR assays using a mollicutes DNA panel

Mollicutes species	Strain	PCR assays^a^						Real-time PCR^b^ melting Temperature (°C)
		1	2	3	4	5	6	
*Acholeplasma (A.) granularum*	BTS-39	−	+	+	+	+	+/−	86.5
*A. laidlawii*	PG8	−	+	+	+	+	+/−	86.5
*A. modicum*	PG49	−	+	+	+	+	+/−	86.5
*A. oculi*	19-L	−	+	+	+	+	+/−	85
*A. parvum*	H23M	−	+	+	+	+	+/−	85
*Mesoplasma florum*	L1	−	+	+	+	+	+	86
*Mycoplasma (M.) agalactiae*	PG2	+	+	+	+	+	+	85.5
*M. alkalescens*	PG51	+	+	+	+	+	+	86
*M. arginini*	G230	+	+	+	+	+	+	86
*M. auris*	CIP 105677	+	+	+	+	+	+	86
*M. bovigenitalium*	PG11	−	+	+	+	+	+	86
*M. bovirhinis*	PG43	+	+	+	+	+	+	87
*M. bovis*	PG45	−	+	+	+	+	+	86
*M. bovoculi*	M165/69	−	+	+	+	+	+	85.5
*M. canadense*	275C	−	+	−	+	+	+	86
*M. capricolum* subsp. *capricolum*	California Kid	+	+	+	+	+	+	87
*M. capricolum* subsp. *capripneumoniae*	F38	−	+	+	+	+	+	86.5
*M. caviae*	G122	−	+	+	+	+	+	86
*M. columbinum*	MMP-1	+	+	+	+	+	+	85.5
*M. columbinasale*	694	+	+	+	+	+	+	85.5
*M. columborale*	MMP-4	+	+	+	+	+	+	86.5
*M. cottewii*	VIS	+	+	+	+	+	+	87
*M. fermentans*	A918 C1	+	+	+	+	+	+	86
*M. flocculare*	Ms42	−	+	+	+	+	+	86.5
*M. gallinaceum*	DD	−	+	+	+	+	+	86
*M. gallinarum*	PG16	−	+	+	+	+	+	86
*M. gallisepticum*	PG31	+	+	+	+	+	+	86
*M. gallopavonis*	WP1	−	+	−	−	−	+	86.5
*M. hominis*	PG21	+	+	+	+	+	+	86.5
*M. hyopharyngis*	H3-6B	+	+	+	+	+	+	85.5
*M. hyopneumoniae*	Ms42	−	+	+	+	+	+	86.5
*M. hyorhinis*	BTS-7	−	+	+	+	+	+	86.5
*M. hyosynoviae*	S16	−	+	+	+	+	+	86
*M. imitans*	4229	+	+	+	+	+	+	86.5
*M. iners*	PG30	+	+	+	+	+	+	85.5
*M. iowae*	695	+	+	+	+	+	+	85
*M. leachii*	PG 50	+	+	+	+	+	+	86
*M. lipofaciens*	ML64	+	+	+	+	+	+	86
*M. maculosum*	PG 15	−	+	+	+	+	+	86
*M. meleagridis*	17,529	−	+	+	+	+	+	86
*M*. *mycoides* subsp. *capri*	Y-GOAT	+	+	+	+	+	+	87
*M. mycoides* subsp*. mycoides* SC	PG1	+	+	+	+	+	+	87
*M. neophronis*	G.A.	+	+	+	+	+	+	86
*M. opalescens*	MH5408	+	+	+	+	+	+	86
*M. phocicerebrale*	D’049	+	+	+	+	+	+	86
*M. phocidae*	105	+	+	+	+	+	+	86
*M. phocirhinis*	DG52	+	+	+	+	+	+	86
*M. sp. Phocoena*	C-269	+	+	+	+	+	+	86.5
*M. pneumoniae*	FH M III	+	+	+	+	+	+	86
*M. pullorum*	CKK	+	+	+	+	+	+	86
*M. putrefaciens*	KS1	+	+	+	+	+	+	87
*M. spumans*	PG13	+	+	+	+	+	+	86
*M. sualvi*	Mayfield B	+	+	+	+	+	+	86
*M. synoviae*	WVU 1853	−	+	+	+	+	+	84.5
*M. verecundum*	107	−	+	+	+	+	+	86
*M. wenyonii* ^c^	Massachusetts	+	+	+	+	+	+	84.5
*M. yeatsii*	GIH	+	+	+	+	+	+	87
*M. zalophi*	CSL 4296	+	+	+	+	+	+	86
*M. zalophidermidis*	4779	+	+	+	+	+	+	86
*Phytoplasma*	PD	NT^d^	NT	NT	NT	NT	NT	84.5^e^
*Phytoplasma*	ESFY	NT	NT	NT	NT	NT	NT	84.5^e^
*Spiroplasma* spp.	−	+	+	+	+	+	+	87
*Ureaplasma urealyticum*	960	+	+	+	+	+	+	85

^a^PCR assays: 1, Hotzel et al. [17]; 2, Spergser et al. [27], 3, McAuliffe et al. [18]; 4, Van Kuppeveld et al. [6]; 5, Botes et al. [28]; 6, Ramírez et al. [16] and real-time PCR application of PCR assays n°4 and n°5

^b^Real-time PCR was performed with primers pairs number 4 and 5, giving both the same result

^c^DNA extracted, identified and donated by Animal and Plant Health Agency (APHA, UK)

+: amplification test positive -: amplification test negative ±: amplification of more than one DNA fragment

^d^NT: Not tested

^e^Phytoplasma DNA extracted, identified and donated by Assunta Bertaccini (University of Bologna) was only tested using Botes et al. [28] real-time assay

Non-mollicutes bacteria used in this study are shown in Table 2. *Bacillus cereus* (ATCC 11778), *Escherichia coli* (ATCC 25922), *Pseudomonas aeruginosa* (ATCC 27853), *Salmonella enterica* (ATCC 13076), *Staphylococcus aureus* (ATCC 33591) and *Streptococcus agalactiae* (ATCC 12386) were obtained from Oxoid (Thermo Scientific) and growth in blood-agar (Pronadisa) at 37 °C for 24 h. In addition, fifteen other bacterial DNA samples were also used. The source of each DNA sample can be seen in Table 2.

**Table 2:** Results of six PCR assays comparison with non-mollicutes bacteria

Non-mollicutes species	Strain	PCR assays^a^						Real-time PCR^b^ melting Temperature (°C)
		1	2	3	4	5	6	
*Acinetobacter baumannii* ^c^	2208	−	+	−	−	−	+	82
*Acinetobacter pleuropneumoniae* ^c^	M62	+	+	−	−	+	±	82.5
*Aeromonas* spp.^c^	−	+	+	+	−	−	±	82.5
*Bacillus cereus* ^d^	ATCC 11778	+	+	+	−	−	±	82.5
*Clostridium* spp. ^c^	−	−	+	−	−	−	±	82
*Erysipelothrix rhusiopathiae* ^c^	a P15	+	+	+	−	−	+	82
*Escherichia coli* ^d^	ATCC 25922	−	−	−	−	−	±	82
*Fusobacterium* spp. ^c^	−	+	+	+	+	+	±	82
*Lactococcus garvieae* ^c^	4976	+	+	+	−	−	+	82.5
*Lactococcus garvieae* ^c^	8831	+	+	+	−	−	+	82.5
*Mycobacterium avium* subsp. *avium* ^e^	CHICKEN AHT1	+	+	+	−	−	+	82.5
*Mycobacterium avium* subsp. *paratuberculosis* ^e^	JC31	±	+	+	−	−	+	83
*Photobacterium damselae* subsp. *piscicida* ^c^	94/99	+	+	+	−	+	±	82.5
*Photobacterium damselae* subsp. *piscicida* ^c^	C2	+	+	+	−	+	±	82.5
*Photobacterium damselae* subsp. *piscicida* ^c^	pp3	+	+	+	−	+	±	82.5
*Pseudomonas aeruginosa* ^d^	ATCC 27853	+	+	+	−	−	+	82
*Salmonella enterica sv enteridis* ^d^	ATCC 13076	+	−	−	−	−	±	82.5
*Staphylococcus aureus* ^d^	ATCC 33591	+	+	+	−	−	±	82
*Streptococcus agalactiae* ^d^	ATCC 12386	+	+	+	−	+	+	82.5
*Streptococcus suis* ^e^	735	+	+	+	−	+	+	82.5
*Vibrio anguillarum* ^c^	Baumann 114	−	+	+	−	+	±	82.5

^a^PCR assays: 1, Hotzel et al. [17]; 2, Spergser et al. [27], 3, McAuliffe et al. [18]; 4, Van Kuppeveld et al. [6]; 5, Botes et al. [28]; 6, Ramírez et al. [16] and real-time PCR application of PCR assays n°4 and n°5

^b^Real-time PCR was performed with assays number 4 and 5, giving both the same result

^c^DNA extracted, identified and donated by Infectious Diseases and Epidemiology unit (Universidad de Las Palmas de Gran Canaria, Spain)

^d^From commercial isolates (Oxoid)

^e^DNA extracted, identified and donated by Animal and Plant Health Agency (APHA, UK)

+: amplification test positive -: amplification test negative ±: amplification of more than one DNA fragment

### DNA extraction and DNA concentration estimation

DNA was extracted from cultures after 48–72 h of incubation. The Realpure Genomic DNA extraction kit (Real) was used to obtain purified genomic DNA and its concentration was quantified with a NanoDrop 1000 Spectrophotometer v.3.7 (Thermo Scientific), in both cases following the manufacturer’s instructions. The integrity of DNA was assessed by electrophoresis in a 1% agarose gel (8 V/cm), precast with Gel Red (Biotium) and analysed with QUANTITY ONE software (Bio-Rad), using Lambda-HindIII Digest (Takara) as molecular weight marker.

### DNA identity confirmation

Identity and purity of mollicutes samples and *Acinetobacter baumannii*, *Aeromonas* spp., *Clostridium* spp., *Fusobacterium* spp. and *Erysipelothrix rhusiopathiae* bacterial DNAs were confirmed by sequencing of the 16S–23S rDNA ISR [16]. For acholeplasmas, only the first set of primers based on the 16S rRNA gene were used, as described before [24]. All sequencing reactions were performed by Macrogen Europe (The Netherlands). The nucleotide sequences were compared to previously described bacterial species deposited in GenBank nucleotide database using the Basic Local Alignment Search Tool (BLASTn).

### Comparison of PCR assays

Six PCR assays (Table 3) were performed using all samples listed in Tables 1 and 2. PCR reactions were performed in triplicate on a Mastercycler Gradient (Eppendorf) using illustra™ PureTaq™ Ready-to-Go™ PCR beads (GE Healthcare), in a final reaction volume of 25 μl that included 1 μl (20 μM) of each forward and reverse primer, 3 μl of genomic DNA and PCR-grade water (Sigma-Aldrich). Each PCR was performed using the primers and protocols described elsewhere (Table 3). Positive and negative controls were used in every PCR run. *M. mycoides* subsp. *capri* (Y-GOAT) DNA was used as positive control. In addition, all PCR reactions were repeated twice using an alternative PCR thermocycler (MyCycler, Bio Rad) to account for the possible variability between different devices.

**Table 3:** Primers for detection of mollicutes

PCR assay	Identification	Sequence (5′-3′)	Amplicon size (bp)	Gene target	Reference
1	Myc23F1729	5′ CTAAGGTDAGCGAGWDAACTAT AG 3’	102–110	23S rRNA	Hotzel et al. [17]
	Myc23R1843	5′ CCCCYCWTSYTTYACTGMGGC 3’			
2	MW28	5′ CCAGACTCCTACGGGAGGCA 3’	580	16S rRNA	Spergser et al. [27]
	MW29	5′ TGCGAGCATACTACTCAGGC 3’			
3	GC341F	5’CGCCCGCCGCGCGCGGCGGGCGGGGCGGGGGCACGGGGGGCCTACGGGAGGCAGCAG 3’	340	16S rRNA	McAuliffe et al. [18]
	R543	5′ ACCTATGTATTACCGCG 3’			
4	GPO3	5′ GGGAGCAAACAGGATTAGATAC CCT 3’	270	16S rRNA	Van Kuppeveld et al. [6]
	MGSO	5′ TGCACCATCTGTCACTCTGTTAACCTC 3’			
5	GPO3F	5′ TGGGGAGCAAACAGGATTAGAT ACC 3’	270	16S rRNA	Botes et al. [28]
	MGSO	5′ TGCACCATCTGTCACTCTGTTAACCTC 3’			
6	16S + C	5′ CGTTCTCGGGTCTTGTACAC 3’	400–600	ISR^a^	Ramírez et al. [16]
	23S–B	5′ CGCAGGTTTGCACGTCCTTCATCG 3’			

^a^ISR: Intergenic spacer region

A 5 μl aliquot of each PCR product was separated by electrophoresis on 1% agarose gels and stained with Gel Red (Biotium). PCR products obtained using the primer set described by Hotzel et al. [17] were resolved in 3% agarose gels. A DNA molecular weight ladder (Amplisize 2000–50 pb, Bio-Rad) was used to estimate the size of PCR products. Positive results were accepted when a single band with the expected size amplicon was observed. All gels were visualized using an image acquisition system (ChemiDoc™ XRS+ System with Image Lab™ Bio-Rad).

### Assay sensitivity and specificity

Analytical sensitivity was performed to determine the detection limit of the assays. For that purpose, serial 10-fold dilutions to 10^−10^ of culture was made with *M. mycoides* subsp. *capri* (Y-GOAT). Genome copy numbers were estimated by using *M. mycoides* subsp. *capri* type strain genome size as reference (GenBank accession number NZ_ANIV00000000.1), using an online application [25].

The detection limit was also calculated with *Acholeplasma* (*A.*) *laidlawii* (PG8). To test for specificity, the different assays were used as described above with the non-mollicutes bacteria listed in Table 2.

The DNA extraction and conventional PCR reaction was carried out on each dilution as explained above. DNA concentration from the undiluted sample was calculated using Qubit® fluorometer (Invitrogen).

The sensitivity, specificity and predictive values of the different PCR assays were calculated, with a 95% of confidence level, using the on-line program Win-Epi 2.0 [26]. It was considered a false positive product when the amplicon had the same size described for each assay (Table 3). When a PCR product had a different size from the expected, was considered as a non-specific product.

### Real-time PCR application

Real-time PCR using the two primers pairs described by van Kuppeveld et al. [6] and Botes et al. [28] and their corresponding PCR protocols were also performed using a MyiQ iCycler Detection System (Bio-Rad). A standardised 25 μl reaction mixture containing 12.5 μl of iQ SYBR Green Supermix (Bio-Rad), 1 μl (20 μM) of each forward and reverse primer, 5 μl of template and PCR-grade water (Sigma-Aldrich) was used. Moreover, the melting peak temperature (Tm) was analysed. After the PCR reaction, a melt curve step was added (from 68 °C, increasing at 0.5 °C/s to 95 °C, with acquisition data every 1 s) to the real-time protocl. The MyiQ system software 1.0.410 (Bio-Rad) was used for data analysis. Melting curves were converted into Tm by plotting the rate of change in fluorescence with temperature versus temperature (−d(RFU)/dT versus T), being RFU, relative fluorescent units. Positive and negative controls used in each real-time PCR run were the same as those used for conventional PCRs. The Tm in this assay was considered as a mollicutes positive result when a value between 84.5–87 °C was observed. In addition, two positive phytoplasmas (PD and ESFY) were also tested.

### External validation

After the initial study was finished, it was decided to perform an external validation in a second laboratory (School of Veterinary Science, University of Liverpool) to account for potential laboratory to laboratory variation. Botes et al. [28] assay at real-time was used in a Roche LightCycler 480 thermocycler using the LightCycler 480 Sybr Green I Master Mix (Roche) and including the following mycoplasmas: *M. alvi* Ilsley^T^, *M. anatis* 1340^T^, *M. anseris* 1219^T^, *M. buteonis* Bb/T2g^T^, *M. cavipharyngis* 117C^T^, *M. cloacale* 383^T^, *M. columbinasale* 694^T^, *M. columbinum* MMP1^T^, *M. columborale* MMP4^T^, *M. corogypsi* BV1^T^, *M. cynos* H 831^T^, *M. falconis* H/T1^T^, *M. felis* CO^T^, *M. gallisepticum* PG31^T^, *M. gateae* CS^T^, *M. genitalium* G-37^T^, *M. glycophilum* 486^T^, *M. gypis* B1/T1^T^, *M. imitans* 4229^T^, *M. iners* PG30^T^, *M. iowae* 695^T^, *M. lipofaciens* R171^T^, *M. meleagridis* 17529^T^, *M. muris* RIII-4^T^, *M. penetrans* GTU-54-6A1^T^, *M. pirum* HRC 70-159^T^, *M. pneumoniae* FH^T^, *M. pullorum* CKK^T^, *M. sphenisci* UCMJ^T^, *M. sturni* UCMF^T^, *M. synoviae* WVU 1853^T^, *M. testudinis* 01008^T^. Mycoplasmas were cultured, identified by immunofluorescence and the DNA was extracted using Chelex resin as previously described [16].

Furthermore, two blood DNA samples from haemoplasma-positive anaemic cats, were analysed. Also, the following non-mollicutes field strain bacteria, isolated and identified by the Diagnostic Laboratory Service (School of Veterinary Science, University of Liverpool), were tested: *Escherichia coli, Pseudomonas aeruginosa, Staphylococcus pseudintermedius, Klebsiella pneumoniae, Acinetobacter baumannii, Bordetella bronchiseptica* and *Streptococcus faecium*. The DNA was extracted from fresh cultures 24 h post inoculation, using QIAamp DNA mini kit (Qiagen) and following the manufacturer’s instructions.

## Results

### PCR assays and real-time PCR application

Mollicutes and non-mollicutes grew normally in their respective media/culture conditions. DNA from cultures was extracted using the method previously described. No false positive or non-specific reactions were observed by conventional PCR after testing all samples included in this work. The results (Tables 1 and 2) obtained with the two conventional thermocyclers were similar. All positive products obtained from conventional PCR were visualised on agarose gels. The amplicons observed exhibited the expected length previously described, ranging in size from 102 to 600 bp (Table 3). Sequences were compared in GenBank nucleotide database, giving similarities results of 99–100% (data not shown), thus confirming their identity.

**Fig. 1:**
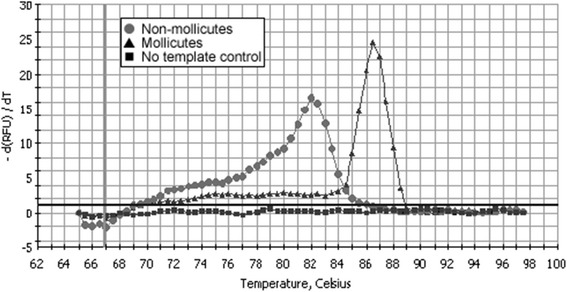
Melting curves for PCR products for the specific detection of mollicutes obtained using Botes et al. assay. Positive samples produced a melting peak between 84 and 87 °C. Negative samples did not produce any melting peak. Unspecific reactions produced a melting peak between 82 and 83 °C. Mollicutes DNA control: *M. hyorhinis*. Non mollicutes DNA control: *Staphylococcus aureus*

Based on the conventional PCR results (Tables 1 and 2), the PCR assays described by van Kuppeveld et al. [6] and Botes et al. [28] were evaluated to be adapted to real-time PCR technology, due to their superior performance characteristics. All mollicutes tested produced positive fluorescence amplification results when tested using both assays adapted to real-time PCR, while all negative controls produced no detectable fluorescence. Non-mollicutes bacteria showed either no fluorescence or an unspecific late fluorescence signal. After amplification, Tm analysis (Fig. 1) allowed us to differentiate between mollicutes and other bacteria. The Tm for mollicutes ranged from 84.5 to 87 °C (Table 1), depending on the mollicutes species, with no correlation to the amount of DNA in the sample. In contrast, the other non-mollicutes samples showed a Tm between 82 and 83 °C (Table 2) or did not show any fluorescence, so the Tm could not be calculated.

### Specificity

Specificity was evaluated in a mollicutes and non-mollicutes set of previously characterised DNA control samples.

The results obtained for the PCR assays applied to mollicutes species are given in Table 1. The PCR described by Hotzel et al. [17] produced the expected amplicon in 38 of the 61 (62.3%) mollicutes tested. The PCR described by McAuliffe et al. [18] managed to detect 59 of the mollicutes tested (96.7%), while van Kuppeveld et al. [6] and Botes et al. [28] conventional PCRs detected 60 out of 61 (98.4%). *M. gallopavonis* was the sample that gave a negative result for the two latter assays. This mycoplasma and *M. canadense* could not be detected by McAuliffe et al. [18] primers. Hotzel et al. assay [17] failed to detect 23 mollicutes, including the two mycoplasmas cited above. However, the DNA samples from these two mycoplasmas were found to have a low concentration of DNA (< 1,5 ng/μl, data not shown). The PCR described by Spergser et al. [27] was successful at detecting all mollicutes (100%). Ramírez et al. [16] PCR produced bands from all samples tested (100%), although it produced two bands when testing acholeplasmas. The real-time applications of van Kuppeveld et al. [6] and Botes et al. [28] could detect all selected mollicutes, giving the same Tm values with both assays. Diagnostic sensitivity values are shown in Table 4.

**Table 4:** Results of the PCR assay against a panel of mollicutes and non-mollicutes bacteria and their sensitivity, specificity, positive and negative predictive values

	1^a^		2		3		4		5		6		Real-time	
	M^b^	B	M	B	M	B	M	B	M	B	M	B	M	B
Test +	38	17	61	19	59	16	60	1	60	8	61	21	61	0
Test -	23	4	0	2	2	5	1	20	1	13	0	0	0	21
Se^c^	62.3% (50.1-74.5)		100.0% (100.0–100.0)		96.7% (92.3–101.2)		98.4% (95.2–101.5)		98.4% (95.2–101.5)		100.0% (100.0–100.0)		100.0% (100.0–100.0)	
Sp^c^	19.0% (2.3- 35.8)		9.5% (−3.0–22.1)		23.8% (5.6–42.0)		95.2% (86.1–104.3)		61.9% (41.1–82.7)		0.0% (0.0–0.0)		100.0% (100.0–100.0)	
PPV^c^	69.1% (56.9-81.3)		76.3% (66.9–85.6)		78.7% (69.4–87.9)		98.4% (95.2–101.5)		88.2% (80.6–95.9)		74.4% (64.9–83.8)		100.0% (100.0–100.0)	
NPV^c^	14.8% (1.4-28.2)		100.0% (100.0–100.0)		91.5% (85.4–97.5)		95.2% (86.1–104.3)		92.9% (79.4–106.3)		-		100.0% (100.0–100.0)	

^a^PCR assays: 1, Hotzel et al. [17]; 2, Spergser et al. [27], 3, McAuliffe et al. [18]; 4, Van Kuppeveld et al. [6]; 5, Botes et al. [28]; 6, Ramírez et al. [16] and real-time PCR application of PCR assays n°4 and n°5

^b^M: mollicutes, n: 61; B: non-mollicutes bacteria, n: 21

^c^Se: Sensitivity; Sp: Specificity; PPV: Positive predictive value; NPV: Negative predictive value

When applied to non-mollicutes bacteria, the PCRs described by Hotzel et al. [17], Spergser et al. [27], McAuliffe et al. [18], Ramírez et al. [16], van Kuppeveld et al. [6], Botes et al. [28], and the real-time PCR applications of the two latter gave false positive results, out of the 21 bacteria analysed, in 17 (81%), 19 (90.5%), 16 (76.2%), 21 (100%), 1 (4.8%), 8 (38.1%) and 0 (0%) samples respectively. No non-specific products were detected. However, Ramírez et al. [16] assay produced multiple bands in nine of the non-mollicutes bacteria tested. Specificity values are shown in Table 4. The four conventional PCRs had specificity values ranging from 0.0% to 95.2%. Both real-time PCRs had a specificity value of 100%, while no false positives results were observed. Occasionally the non-mollicutes bacteria tested produced melting peaks. However, the Tm of these peaks was always below 83 °C, as described above.

### Analytical sensitivity

Detection limits were calculated using serial dilutions of *Mycoplasma mycoides* subsp. *capri* (Y-GOAT) and *A. laidlawii* PG8 (Table 5). Spergser et al. [27] assay showed the highest analytical sensitivity using the conventional PCR format, being able to detect a dilution of 10^−5^, equivalent to approximately 15 genome copies of *M. mycoides* subsp. *capri* per reaction, based on our initial DNA concentration (1.72 ng/μl of DNA).

**Table 5:** Results of the detection limits of the primers pairs against DNA serial dilutions of *Mycoplasma mycoides* subsp. *capri* (Y-Goat) and *Acholeplasma laidlawii* (PG8)

Species	PCR assays						Real-time	
	1	2	3	4	5	6	4	5
Y-Goat	10^−1^	10^−5^	10^−2^	10^−2^	10^−3^	10^−3^	10^−4^	10^−5^
PG8	-	10^−5^	10^−2^	10^−2^	10^−3^	10^−3^	10^−4^	10^−5^

^a^1: Hotzel et al., [17]; 2: Spergser et al., [27]; 3: McAuliffe et al. [18]; 4: van Kuppeveld et al. [6]; 5: Botes et al. [28]; 6 Ramírez et al. [16] and the real-time PCR application of PCR assay n°4 and n°5

Conversely, the PCR assay published by Hotzel et al. [17], targeting the 23S rDNA gene, showed the lowest sensitivity, with a limit of detection 10^−1^ dilution. In addition, this assay was not able to amplify any *A. laidlawii* (PG8) dilutions.

When real-time assays were implemented, there was a decrease in the detection limits in two ten-fold dilutions, down to 10^−4^ and 10^−5^ (equivalent to 150 to 15 genome copies per reaction) for van Kuppeveld et al. [6] and Botes et al. [28] PCR assays, respectively.

It should be noted that in some cases nanodrop-based DNA quantification can slightly underestimate the amount of DNA in the sample, therefore the actual number of genome copies detected could be slightly higher than the number reflected above.

### Positive and negative predictive values

Positive and negative predictive values are shown in Table 4. The PCR assays with the best predictive results were both van Kuppeveld et al. [6] and Botes et al. [28] assays when adapted to real-time technology, showing a 100% of positive and negative predictive values.

### External validation results

External validation gave similar results as before, with a slight decrease in the Tm values. Mollicutes gave Tm values between 82 and 85.5 °C, while bacteria gave Tm below 80 °C or no peak at all, while the negative control showed no detectable fluorescence. Both haemoplasma samples gave a fluorescence peak around 82.5 °C.

## Discussion

In this study, six conventional PCR assays previously described to be able to detect members of the Class *Mollicutes* [6, 16–18, 27, 28] were compared. A comprehensive panel of 61 mollicutes (Class *Mollicutes*, Phylum *Tenericutes*) was used, representing four out of five of the mollicutes phylogenetic groups described (anaeroplasmas, mesoplasmas, spiroplasmas and mycoplasmas) [1]. Mollicutes are evolutionarily related to certain clostridia [1]. For this reason, twenty-one bacterial DNAs, including species closely related to the mollicutes, were also used to evaluate the specificity of the PCR reactions.

For conventional PCR, Spergser et al. [27] assay gave the highest sensitivity for the mollicutes sample set (100%), while the lowest (62,3%) was given by the Hotzel et al. [17] assay. All PCR assays could detect most of the mollicutes tested. Acholeplasmas and *Me. florum* were also detected by all PCR assays apart from Hotzel et al. [17] assay. The latter result could be related to the lack of analytical sensitivity, presenting the highest detection limit (10^−1^) and not to a lack of specificity. This could also apply to false negatives in other assays, because the concentration of DNA in these samples was demonstrated to be low (< 1.5 ng/μl). When specificity was tested using non-mollicutes bacteria, it varied from 95.2% as observed using van Kuppeveld et al. [6] assay, to 0.0% [16].

McAuliffe et al. [18] primers had been previously used with denaturing gradient gel electrophoresis (DGGE) to enable rapid identification of many mycoplasma species. In our study, amplicons were visualised in agarose gels. This is likely to explain the low sensitivity found when testing this assay. We found that Spergser et al. [27] primer set, although very unspecific, had the advantage of obtaining amplicons from a small amount of template, which could be useful to identify fastidious mollicutes. However, when these primers were used for DNA sequence analysis, limited 16S rDNA sequence was obtained. This disadvantage could be overcome by using the primer sets described by Ramírez et al. [16]. This assay was found to be very useful for sequencing the complete ISR and was used as quality control in our study. However, this primer set is also unspecific and less sensitive than others sets evaluated in this work.

Van Kuppeveld et al. [6] assay proved to be the best preforming conventional PCR test based on our data, showing the highest specificity (95.2%) and positive predictive value (98.4%), while its sensitivity was also high (98.4%). The primer set could detect nearly all the mollicutes evaluated but *M. gallopavonis*. This negative result could be related to the small amount of DNA of the sample, having a concentration of less than 1.5 ng/μl of DNA.

Our study agrees with van Kuppeveld et al. [6], confirming that the PCR primer set described can detect mycoplasmas, acholeplasmas, ureaplasmas and spiroplasmas, in addition to mesoplasmas (*Me. florum*), and haemoplasmas (*M. wenyonii*), as found in our study. Using this assay, a false positive result for *Fusobacterium* spp. was detected; a cross-reaction that was previously described by Jensen et al. [29]. In our study, this PCR assay was chosen for further optimization, applying it into real-time PCR. In addition, Botes et al. [28] assay was also optimised for real-time PCR, due to its similar performance characteristics.

After optimisation, specificity and sensitivity of both assays was improved. However, Botes et al. assay [28] performed slightly better, with equal specificity to van Kuppeveld assay [6] and higher sensitivity. The Tm for mollicutes ranged from 84 to 87 °C. Some bacteria, as well as *Fusobacterium* spp., sometimes gave some unspecific results, with Tm values between 82 and 83 °C, which were considered negative results. Both assays showed their detection limits reduced when compared to conventional PCR assays by two ten-fold dilutions.

The use of ready-to-use PCR beads was chosen to work under comparable conditions, so assay to assay variability was reduced. The targets, 16S rRNA gene, 23S rRNA gene and ISR, have similar number of copies in the genome, ranging from one to three. Botes et al. [28] primer set has nearly the same sequence as van Kuppeveld et al. [6] with a slight modification in the forward primer, having moved the location of the primer two base pairs upstream in the 16S rDNA gene sequence. The increase in sensitivity (10-fold higher) could be explained by the improvement on the forward primer design. He et al. [30] found that the primers were decisive for PCR sensitivity and that testing several primer pairs with slightly differences was very useful in optimizing the sensitivity of PCR, although it is impossible to predict the result in most of the cases.

Real-time PCR has several advantages over conventional PCR [11]. In our study a 100-fold higher analytical sensitivity was obtained in the application of the real-time PCR for the van Kuppeveld et al. [6] and Botes et al. [28] assays.

Our real-time PCR applications were coupled with Tm analyses to determine the presence of non-specific amplification products. Using Tm analysis, we could differentiate between mollicutes (Tm range: 84.5–87 °C) and non-mollicutes (Tm range: 82–82.5 °C). A similar strategy was used by Baczynska et al. [11] in the development of a real-time PCR for detection of *M. hominis*, when samples with a Tm below 61 °C were considered as negative results.

Sung et al. [14] found that the currently available procedures were not enough for the simultaneous detection of the major mycoplasmas. In addition, a bigger effort must be put into assay validation, with a focus on specificity that should be always performed on large panels of well-characterized samples [29]. In this study, 61 mollicutes and 21 non-mollicutes have been tested. When this panel of samples was used under real-time conditions, a 100% sensitivity, specificity and predictive values were achieved. In our study, we decided to perform an external validation to Botes et al. [28] real-time assay, because the selection of convenience samples and the lack of blinding in the evaluation of the assays could have led to overoptimistic sensitivity and specificity estimates [31]. This external validation was done in a second laboratory using different conditions to those previously described, including a different DNA extraction method, thermocycler and Sybr Green master mix. The results of our post analysis are consistent with the original data, with the difference being that the Tm values dropped by a mean of 2.5 °C. Some degree of variation in the Tm was expected as various factors could affect the outcome of the real-time assay. The Tm of a PCR product depends mainly on its length, GC content and sequence. However, melting behaviour of PCR products can be affected by Sybr Green I concentration and the temperature transition rates. Ririe et al. [32] found that Tm of PCR products increased by up to 3 °C as the heating rate increases. Also, the melting curve shifts to higher temperatures when the Sybr Green I concentration was increased. The difference in the outcome of the study and the post analysis results could be explained by this fact. Therefore, for reproducible melting curves it is recommended to control the Sybr Green I concentration, the ramp rate and the salt concentration [32], as well as the use of proper controls in each run. Ririe et al. [32] also described that Tm values differing by 2 °C can be easily distinguished within mixtures. We have found the same evidence in our study, where the differences in the Tm values between mollicutes and non-mollicutes were found to be at least 2 °C apart. For that reason, the real-time application could be useful not only for pure cultures, but also for mixed cultures and clinical samples. As the real-time assays tested used generic mollicutes primers, they will not be limited to the detection of specific species, but to any mollicutes present in the sample tested. Real-time based assays are likely to be valuable for detecting novel and unknown species of mollicutes. These assays could also be useful as a screening tool for finding mollicutes in clinical samples, cultures and cell cultures.

## Conclusions

The use of generic real-time PCR was demonstrated to be a useful technique for the rapid detection of mollicutes. Van Kuppeveld et al. [6] and Botes et al. [28] real-time PCR assays could detect mycoplasmas, acholeplasmas, mesoplasmas, hemoplasmas, spiroplasmas and ureaplasmas in a highly sensitive and specific manner. Phytoplasma could also be detected using Botes et al. assay [28]. Melting peak temperature analysis helped to differentiate between mollicutes and non-mollicutes microorganisms. We consider that the real-time PCR assays described in this paper could be of great use for screening cell line contaminants, clinical samples as well as detecting non-culturable, unknown species of mollicutes or mollicutes whose growth is slow or difficult.

## Abbreviations

ATCC: American Type Culture Collection
DNA: Deoxyribonucleic acid
NAT: Nucleic acid amplification test
PCR: Polymerase chain reaction
rRNA: Ribosomal ribonucleic acid
Tm: Melting peak temperature
